# RCAN1.4 mediates high glucose-induced matrix production by stimulating mitochondrial fission in mesangial cells

**DOI:** 10.1042/BSR20192759

**Published:** 2020-01-17

**Authors:** Hong-Min Chen, Jia-Jia Dai, Rui Zhu, Xue-Yu Sang, Fang-Fang Peng, Hong Yu, Bai-Fang Zhang

**Affiliations:** Department of Biochemistry and Hubei Provincial Key Laboratory of Developmentally Originated Disease, Wuhan University School of Basic Medical Sciences, Wuhan, P.R. China

**Keywords:** extracellular matrix protein, mesangial cell, mitochondrial fission, RCAN1.4

## Abstract

High glucose (HG)-induced mitochondrial dynamic changes and oxidative damage are closely related to the development and progression of diabetic kidney disease (DKD). Recent studies suggest that regulators of calcineurin 1 (RCAN1) is involved in the regulation of mitochondrial function in different cell types, so we investigate the role of RCAN1 in mitochondrial dynamics under HG ambience in rat glomerular mesangial cells (MCs). MCs subjected to HG exhibited an isoform-specific up-regulation of RCAN1.4 at both mRNA and protein levels. RCAN1.4 overexpression induced translocation of Dynamin related protein 1 (Drp1) to mitochondria, mitochondrial fragmentation and depolarization, accompanied by increased matrix production under normal glucose and HG ambience. In contrast, decreasing the expression of RCAN1.4 by siRNA inhibited HG-induced mitochondrial fragmentation and matrix protein up-regulation. Moreover, both mitochondrial fission inhibitor Mdivi-1 and Drp1 shRNA prevented RCAN1.4-induced fibronectin up-regulation, suggesting that RCAN1.4-induced matrix production is dependent on its modulation of mitochondrial fission. Although HG-induced RCAN1.4 up-regulation was achieved by activating calcineurin, RCAN1.4-mediated mitochondrial fragmentation and matrix production is independent of calcineurin activity. These results provide the first evidence for the HG-induced RCAN1.4 up-regulation involving increased mitochondrial fragmentation, leading to matrix protein up-regulation.

## Introduction

Diabetic kidney disease (DKD) is one of the major microvascular complications of diabetes mellitus and an important cause of death in end-stage renal disease [1,2]. Various clinical strategies, including glucose control, angiotensin-converting enzyme inhibitors and angiotensin II receptor antagonists, only delay the progress of DKD. The development of novel therapeutic agents is thus an important goal [3].

Growing evidence suggests that high glucose (HG)-induced mitochondrial injury, especially mitochondrial dynamic changes and oxidative damage is closely related to the occurrence and development of DKD [4–6]. Mitochondria are highly dynamic organelles, which constantly undergo fusion and fission, changing the shape, size and intracellular distribution, in order to maintain cell homeostasis and viability [7,8]. Mitochondrial dynamics is an important quality control process, regulated by a set of proteins including fusion-related protein mitofusin 1/2 (Mfn1/2) and optic atrophy 1 (Opa1), fission-related protein dynamin related protein 1 (Drp1) and mitochondrial fission 1 protein (Fis1) [9,10]. Among them, Mfn1 and Mfn2 mainly regulate the fusion of mitochondrial outer membrane (MOM), and Opa1 is mainly involved in the fusion of mitochondrial inner membrane and crista. Mitochondrial profission protein Drp1 is mainly present in the cytoplasm. The translocation of Drp1 to the outer membrane receptors of mitochondria in the form of small oligomers is considered to be the necessary initial step of mitochondrial fission [9,10]. Fis1 can combine with Drp1, which accelerates mitochondrial fission when overexpressed [11]. Abnormal mitochondrial dynamics and mitochondrial dysfunction could result in renal cell injury, proteinuria and loss of renal functions [4–6] in DKD. However, the mechanisms of dynamic regulation and mitochondrial damage in hyperglycemia environment remain to be defined.

Regulator of calcineurin 1 (RCAN1) is initially referred to as Down Syndrome Critical Region 1 (DSCR1), located in the region 21q22.1-q22.2 of human chromosome 21. It was first found to involve the pathogenesis of Down syndrome [12]. As an endogenous regulator of calcineurin, RCAN1 plays a key role in cell differentiation, migration and apoptosis [13–15]. RCAN1 gene contains seven exons and can produce different subtypes by variable splicing. The dominant subtypes are RCAN1.1 encoded by exon 1 and RCAN1.4 encoded by exon 4. Although RCAN 1.1 and RCAN 1.4 have the same C-terminal domain and contain 168 amino acids encoded by exons 5, 6 and 7 [15], they may have different expression patterns, different regulation mechanisms and independent functions [16]. RCAN1.1 seems to be constitutively expressed in most tissues, while transcription of RCAN1.4 is regulated by several stimuli including calcineurin activation [15]. The expression and distribution of RCAN 1.1 and RCAN 1.4 in kidney tissues are still unclear, and the role of RCAN1 in DKD is not clearly defined. It has been reported that RCAN1.1 level is down-regulated in the glomeruli of both diabetic patients and mice; moreover, HG decreases the expression of RCAN1.1 in podocytes through epigenetic mechanism [17]. However, Jang et al. demonstrate that RCAN1.1 mRNA level is unchanged in the kidney cortex of the db/db mice [18]. In addition, the mRNA level of RCAN 1.4 is increased in the glomeruli of diabetic mice, and RCAN 1.4 overexpression can up-regulate the mRNA level of collagen I/III in mesangial cells [18], although the exact mechanism is unknown.

Recent studies indicate that RCAN1 is involved in the regulation of mitochondrial function. Increased RCAN1 expression results in impaired mitochondrial function in β cells and neuronal cells [19,20], but high RCAN1 in cardiomyocytes generates a more fused mitochondrial network and enhances mitochondrial function [21]. These contradictory results suggest that the functions of RCAN1 related to the regulation of mitochondrial function appear to be dependent on cell type and the type of stimuli. Whether RCAN1 enhances or impairs mitochondrial function in renal cells remains to be addressed. The link between mitochondria and renal fibrosis in DKD has been recently demonstrated [22]. Mitochondrial dysfunction is associated with TGF-β and angiotensin II-induced extracellular matrix (ECM) accumulation in DKD [23,24]. We therefore sought to investigate whether RCAN1.1 and RCAN1.4 are involved in HG-induced abnormal mitochondrial dynamics and mitochondrial dysfunction in mesangial cells. Furthermore, we explore whether abnormal mitochondrial dynamics mediate increasing matrix production under HG ambience. Elucidating the interaction of RCAN1-driven abnormal mitochondrial dynamics and matrix production can broaden the understanding of glomerulosclerosis pathway and provide a potential drug target for prevention and treatment of DKD.

## Materials and methods

### Culture of mesangial cells (MCs) and treatments

Primary rat MCs (passages 6–18) were cultured in DMEM (Invitrogen, Carlsbad, U.S.A.) with normal glucose (5.6 mM) containing 20% fetal bovine serum (Invitrogen) in the presence of 100 U/ml penicillin and 100 μg/ml streptomycin under an atmosphere of 5% CO_2_ at 37°C. Confluent cells were incubated with either 24.4 mM glucose or 24.4 mM mannitol used as osmotic control. In subsequent experiments, drugs include Mdivi-1, 50 μM for 24 h; MitoTEMPO, 30 μM for 2 h; Cyclosporine A (CsA), 1 μM for 24 h; FK506, 10 nM for 24 h (all purchased from Sigma-Aldrich, St. Louis, U.S.A.).

### RT reaction and real-time quantitative PCR

Total RNA from rat MCs was extracted using TRIzol reagent (Invitrogen) following the manufacture’s recommended conditions. After treatment with DNase I, 2 μg of the RNA was reversely transcribed into cDNA using the PrimerScript RT Enzyme Mix I System (TaKaRa, Dalian, China). Real-time PCR was performed using 2 μl of the synthesized cDNA on the SYBR Green Realtime PCR Master Mix system (TOYOBO, Osaka, Japan) to quantify relative mRNA levels of RCAN1.1 and RCAN1.4. RCAN1.1 sense primer is 5′-GACCCGCGCGTGTTC-3′, and the antisense primer is 5′-TGTCATATGTTCTGAAGAGGGATTC-3′. RCAN1.4 sense primer is 5′-TGCTTGTGTGGCAAACGATG-3′, and the antisense primer is 5′-AGGAACTCGGTCTTGTGCAG-3′. The expression level was normalized to GAPDH level in the same sample (sense, 5′-TGCACCACCAACTGCTTAGC-3′; antisense, 5′- GGCATGGACTGTGGTCATGAG-3′).

### Subcellular fractionation

Mitochondria were isolated using a mitochondria isolation kit (Beyotime Biotechnology, Shanghai, China) according to manufacturer’s instructions. Briefly, MCs were suspended using 1 ml mitochondrial isolation reagent, and then broken by 10% ultrasound power for three times. After centrifugation at 4°C for 10 min at 1000 ***g*** to remove unbroken cells and nuclear pellet, the supernatant was centrifuged at 3500 ***g*** 4°C for 10 min for separation of the mitochondrial pellet from cytosolic fraction. The pellet was suspended gently with 200 μl of mitochondrial storage solution and centrifuged at 3500 ***g*** for 10 min at 4°C. Then, the pellet was suspended gently with 100 μl of mitochondrial lysate to collect mitochondrial fraction.

For whole-cell lysate, MCs were lysed in lysis buffer as described previously [25]. After centrifugation at 12,000 ***g*** for 10 min at 4°C, protein quantification of supernatant was performed by the Bicinchoninic acid method.

### Western blots

Total protein, cytoplasmic protein and mitochondrial protein were separated by SDS-PAGE and transferred to nitrocellulose membranes (Millipore) followed by blocking and immunoblotting with various primary antibodies, including anti-RCAN1, anti-FLAG (Sigma), anti-COX4, anti-Drp1, anti-Fis1, anti-Mfn2, anti-Opa1 (all Cell Signaling Technology, Boston, U.S.A.), anti-fibronectin (Millipore), anti-collagen I, anti-β-tubulin, anti-β-actin (Santa Cruz Biotechnology, Dallas, U.S.A.). After overnight incubation at 4°C, the membranes were immersed in a solution including appropriate secondary antibodies (Santa Cruz) for 1 h at room temperature. The blots were developed using ECL kit (Millipore).

### Plasmid construction and transfection

A full-length human homologue of RCAN1.4 was amplified from a cDNA library (Promega) and subcloned into 3×FLAG-tagged pLHCX retrovirus plasmid (Clontech Laboratories, CA, U.S.A.). Rat MCs were transfected with RCAN1.4 or empty vector using Lipofectamine 3000 kit (Invitrogen) at approximately 60% confluency. Stable transfectants were selected with puromycin containing media for 1 week.

### Assay of calcineurin activity

Calcineurin activity was measured using the calcineurin activity assay kit (Nanjing Jiancheng Bioengineering Institute, China) according to the manufacturer’s protocol. Cell lysates of MCs were collected, and protein concentration was determined by the Bradford assay. The calcineurin activity assay uses RII phosphopeptide substrate with liberated phosphate detected after completion of the reaction using a malachite green reagent. Enzyme activity was calculated from the rate of change of the absorbance at 636 nm (*n* = 3 for each group, assayed in duplicate for each enzyme activity). One micromol inorganic phosphorus per milligram of protein per hour is specified as one unit of calcineurin activity.

### RNA interference and shRNA transfection

An siRNA targeting RCAN1.4 mRNA or negative control were purchased from RioBio (Wuhan, China). Subconfluent rat MCs were transfected with 10 nM siRNA using Lipofectamine RNAiMAX kit (Invitrogen) following the manufacturer’s instructions. The siRNAs used for knockdown experiments were as follows: negative control, sense, 5′-UUCUCCGAACGUGUCACGUTT-3′, antisense, 5′-ACGUGACACGUUCGGAGAATT-3′; rat RCAN1.4, sense, 5′-GAUGAUGUCUUCAGCGAAAUU-3′, antisense, 5′-UUUCGCUGAAGACAUCAUCUU-3′.

Subconfluent rat MCs were transfected with control shRNA or Drp1 shRNA (Santa Cruz) using Lipofectamine 3000 kit (Invitrogen) according to the manufacturer’s instructions. Changes in protein levels of RCAN1.4 or Drp1 were assessed by Western Blot 36 h post-transfection.

### Assessment of mitochondrial morphology

MCs were incubated with 100 nM Mitotracker-Green (Invitrogen) at 37°C for 30 min and observed by confocal microscopy. After paraformaldehyde fixation and permeabilization by detergent, the shape of mitochondria was assessed by randomly selecting 10 fields of cells from different groups (>100 cells per group).

### Flow cytometry analysis of mitochondrial membrane potential (Δ*Ψ*m) and mitochondrial reactive oxygen species (mtROS)

Δ*Ψ*m and mtROS levels were measured with tetramethylrhodamine methyl ester (TMRM, Sigma, 1 μM, 15 min; excitation/emission 543/580 nm) or MitoSOX Red (Invitrogen, 1 μM, 10 min; excitation/emission 510/590 nm). Mitochondrial uncoupler carbonyl cyanide m-chlorophenylhydrazone (CCCP, 30 μM) for 30 min was used as positive control for the Δ*Ψ*m measurement. Afterward, MCs were digested by trypsin for 3 min, and fluorescence was analyzed on a FACScan flow cytometer (Becton Dickinson, Franklin Lakes, U.S.A.). Data were presented by histograms representing the mean fluorescence intensity.

### Determination of ATP content

ATP content in rat MCs was determined using a luciferin/luciferase-based assay (Promega) according to manufacturer’s instructions. Briefly, MCs were cultured on a 96-well plate, and ATP detection reagent was added to medium at volume ratio of 1:1. The liquid was mixed for 2 min on the plate shaker, put at room temperature for 10 min, and then luminescence signal was detected with a multifunctional microplate reader.

### Statistical analysis

Repetition times of independent experiments (*n*) were given in figure legends. Data were reported as means ± SEMs. The differences among multiple groups were analyzed using one-way ANOVA with Newman–Keuls post hoc analysis. The difference was statistically significant when *P* < 0.05. All data were analyzed by SPSS 17.0 software.

## Results

### HG-induced RCAN1.4 up-regulation mediates mitochondrial fission and reduces mitochondrial function in rat MCs

**Figure 1:**
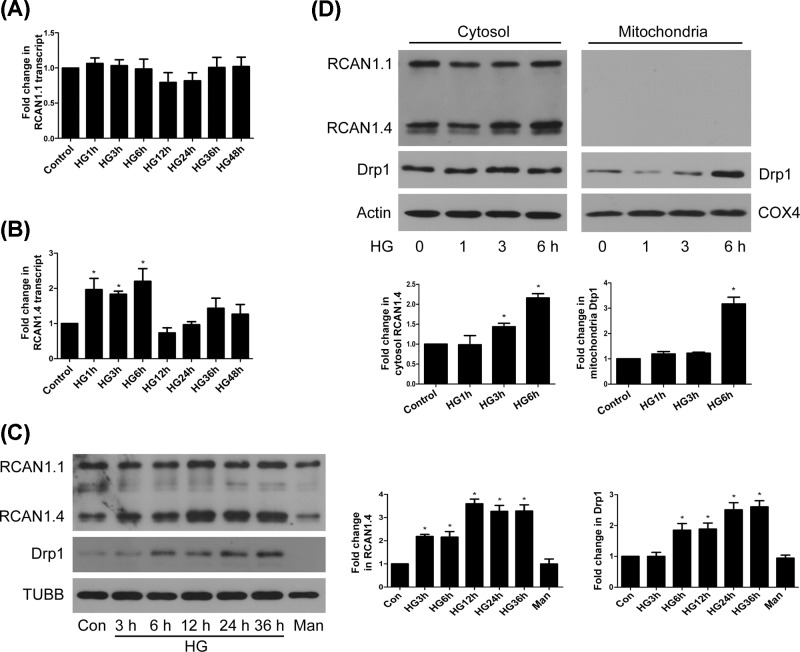
HG induced an isoform-specific up-regulation of RCAN1.4 in rat MCs (**A** and **B**) Rat MCs were treated with 30 mM HG for indicated duration. mRNA levels of RCAN1.1 and RCAN1.4 were detected by real-time RT-PCR with GAPDH as an internal control (*P < 0.05 vs. control, *n* = 4). (**C**) Rat MCs were treated with 30 mM HG for indicated duration. Protein levels of RCAN1 and Drp1 were detected by Western blots, with mannitol for 36 h as a high osmotic control (*P < 0.05 vs. control, *n* = 3). (**D**) MCs were incubated with HG for indicated time, and the protein levels of RCAN1 and Drp1 in cytosol (actin used as loading control) or mitochondria (COX4 used as loading control) were detected by Western blots (*P < 0.05 vs. control, *n* = 3).

The expression of RCAN1.1 can be down-regulated by HG in podocytes [17], while others have demonstrated that 48 h of HG treatment had no effect on mRNA levels of RCAN1.4 in mouse MCs [18]. It is not clear whether short-term treatment with HG affects transcription level of RCAN1, and whether HG has an impact on protein levels of RCAN1.1 and RCAN1.4 in MCs. Our results showed that mRNA and protein levels of RCAN1.1 did not change in response to HG stimulation (Figure 1A,C). Different from RCAN1.1, the mRNA level of RCAN1.4 increased at 1 h, peaked at 6 h, and then went back to the basic level at 12 h. Despite an upward trend after 24 h, there was no significant difference from the control (Figure 1B). As seen in Figure 1C, RCAN1.4 protein was expressed at lower levels than RCAN1.1 in rat MCs, and protein level of RCAN1.4 began to increase at 3 h and lasted for 36 h. Either RCAN1.1 or RCAN1.4 protein level was not affected by high mannitol treatment for 36 h. These results indicated that HG could induce rapid up-regulation of RCAN1.4 isoform at both mRNA and protein levels in MCs.

**Figure 2:**
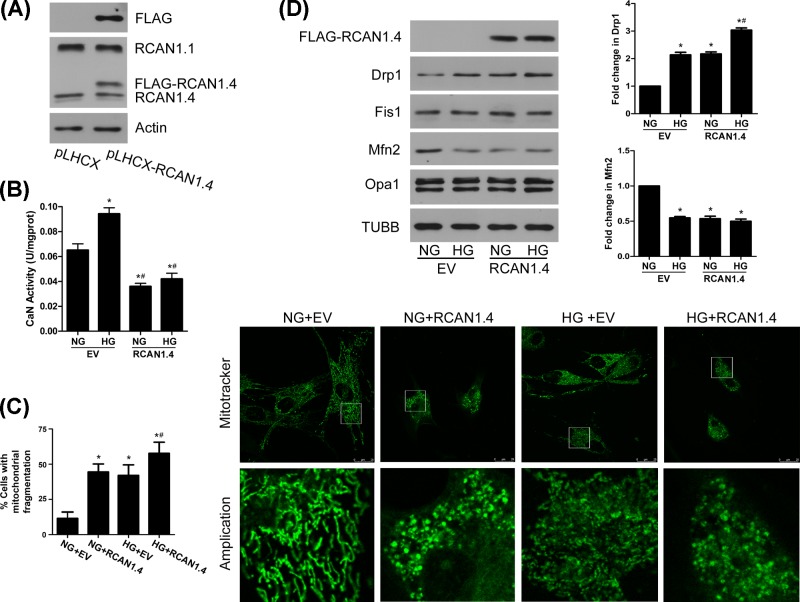
Enhancing effect of RCAN1.4 overexpression on altered mitochondrial dynamics and associated proteins under HG conditions (**A**) MCs were transfected by 3×FLAG pLHCX-RCAN1.4, and protein level of RCAN1.4 was assayed by Western blots. (**B** and** C**) MCs were incubated with normal glucose (NG) or HG for 12 h after empty vector (EV) or RCAN1.4 transfection. Calcineurin activity (**B**) was assayed (*n* = 3), and mitochondrial morphology (**C**) was assessed by Mitotracker-Green staining (*P < 0.05 vs. NG+EV, ^#^*P* < 0.05 vs. HG+EV). (**D**) MCs were incubated with NG or HG for 24 h with or without RCAN1.4 overexpression. The protein levels of Drp1, Fis1, Mfn2 and Opa1 were assayed by Western blots with β-tubulin (TUBB) as loading control (*P < 0.05 vs. NG+EV, ^#^*P* < 0.05 vs. HG+EV, *n* = 4).

**Figure 3:**
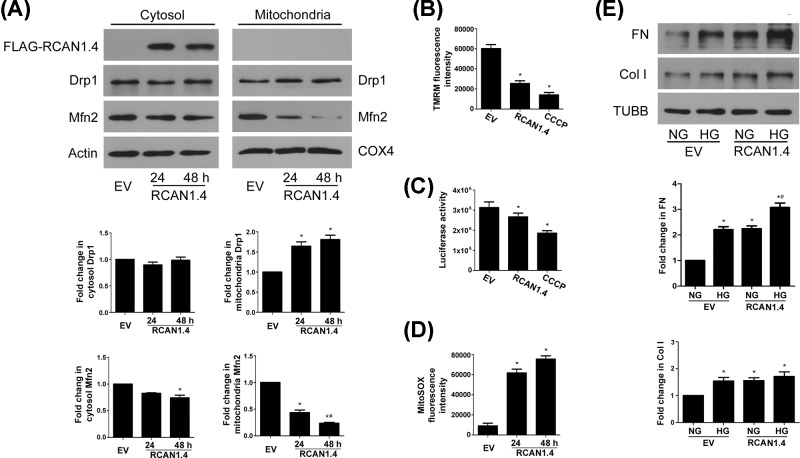
Enhancing effect of RCAN1.4 overexpression on altered Δ*Ψ*m, ATP, ROS generation and matrix production upon HG exposure (**A**) MCs were transfected by RCAN1.4 for 24 and 48 h, and the protein levels of Drp1 and Mfn2 in cytosol or mitochondria were detected by Western blots (*P < 0.05 vs. EV, ^#^*P* < 0.05 vs. RCAN1.4^24 h^, *n* = 3). (**B**) MCs were transfected by RCAN1.4 for 24 h, and Δ*Ψ*m was evaluated by TMRM staining using flow cytometry, with mitochondrial uncoupler CCCP (30 μM for 30 min) as positive control (*P < 0.05 vs. EV, *n* = 3). (**C**) MCs were transfected by RCAN1.4 for 24 h, and ATP content was determined using a luciferin/luciferase-based assay (*P < 0.05 vs. EV, *n* = 4). (**D**) MCs were transfected by RCAN1.4 for 24 and 48 h, and mtROS was evaluated by MitoSOX Red staining using flow cytometry (*P < 0.05 vs. EV, *n* = 3). (**E**) MCs were incubated with NG or HG for 24 h with or without RCAN1.4 overexpression. The protein levels of FN and Col I were assayed by Western blots (*P < 0.05 vs. NG+EV, ^#^*P* < 0.05 vs. HG+EV, *n* = 4).

We next focused on mitochondrial profission protein Drp1 and found that Drp1 was up-regulated at 6 h in response to HG and lasted to 36 h (Figure 1C). Although RCAN1.1 and RCAN1.4 were not translocated to mitochondria, HG-induced up-regulation of Drp1 and translocation to mitochondria is later than the up-regulation of RCAN1.4 protein (Figure 1C,D), indicating that RCAN1.4 may serve as an upstream factor that regulates mitochondrial fission by Drp1 translocation. We then used FLAG-tagged RCAN1.4 overexpression (Figure 2A) to confirm it. Since RCAN1 is an endogenous regulator of calcineurin, we determined calcineurin activity and found that RCAN1.4 overexpression inhibited the calcineurin activity under normal glucose (NG) or HG ambience (Figure 2B). As shown in Figure 2C, the MCs transfected with RCAN1.4 have higher percentages of fragmented mitochondria under NG or HG conditions. We then examined whether RCAN1.4 regulates the expression of mitochondrial dynamics-associated proteins. Although there was not significant change in the expression of Fis1 and Opa1, the protein level of Drp1 was increased, and Mfn2 protein was reduced in MCs overexpressing RCAN1.4 under NG or HG conditions (Figure 2D). This was accompanied with increased translocation of Drp1 to mitochondria, whereas the level of Mfn2 was down-regulated in mitochondria (Figure 3A).

To further investigate whether mitochondrial function is altered by RCAN1.4 overexpression, the fluorescent mitochondrial membrane potential (Δ*Ψ*m) marker, TMRM, was used to evaluate Δ*Ψ*m. TMRM staining showed a loss of Δ*Ψ*m in MCs overexpressing RCAN1.4 (Figure 3B and Supplementary Figure S1A). Consist with this, RCAN1.4 overexpression resulted in decreased intracellular ATP content (Figure 3C) in MCs.

### RCAN1.4 overexpression induces mitochondrial ROS generation and matrix production in rat MCs

Mitochondria are the major source of ROS that are generated as a byproduct of electron transport and oxidative phosphorylation. It has been previously demonstrated that mitochondrial fission is associated with increased mitochondrial ROS (mtROS) level in various cell types [26,27]. We thus investigated the effect of RCAN1.4 on mtROS generation using MitoSOX Red staining and found that mtROS level was increased in MCs overexpressing RCAN1.4 (Figure 3D and Supplementary Figure S1B).

It has been reported that RCAN 1.4 overexpression can up-regulate the mRNA level of collagen I/III in mouse MCs [18]. We next examined whether RCAN1.4 overexpression has any effect on matrix protein production. Western blot analysis showed that the protein levels of fibronectin (FN) and collagen I (Col I) were up-regulated in rat MCs overexpressing RCAN1.4 under NG or HG ambience (Figure 3E).

### RCAN1.4 knockdown attenuates HG-induced mitochondrial fission and matrix production

**Figure 4:**
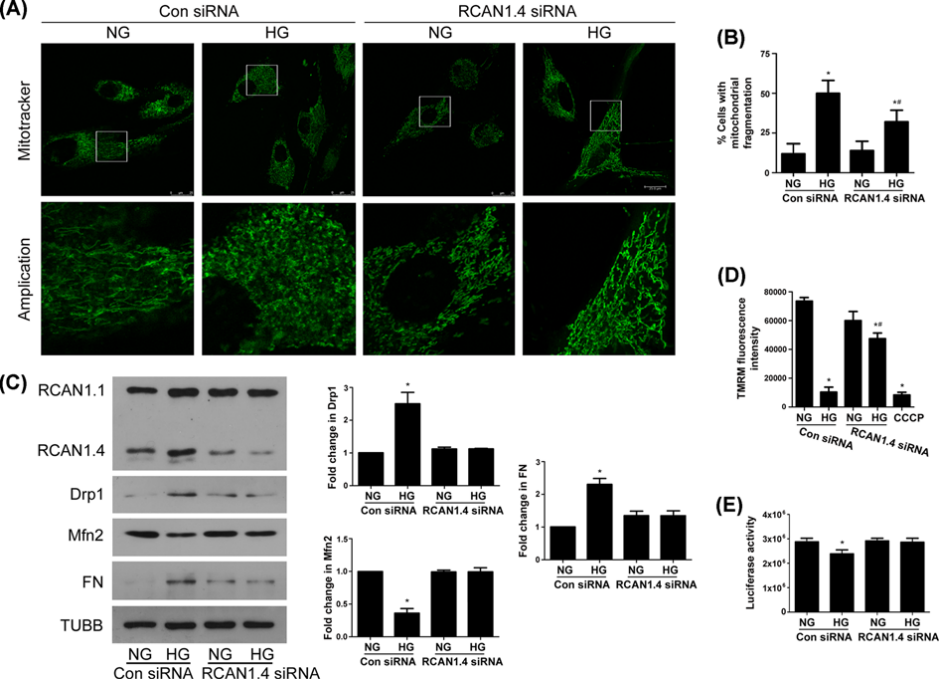
Attenuation of mitochondrial fission, loss of Δ*Ψ*m and increased matrix production after RCAN1.4 knockdown under HG conditions After control or RCAN1.4 siRNA transfection for 36 h, MCs were incubated with NG or HG for 24 h. (**A** and **B**) Mitochondrial morphology was assessed by Mitotracker-Green (*P < 0.05 vs. NG+control siRNA, ^#^*P* < 0.05 vs. HG+control siRNA). (**C**) The protein levels of Drp1, Mfn2 and FN were assayed by Western blots (*P < 0.05 vs. NG+control siRNA, *n* = 3). (**D**) Δ*Ψ*m was evaluated by TMRM staining, with CCCP as positive control (*P < 0.05 vs. NG+control siRNA, ^#^*P* < 0.05 vs. HG+control siRNA, *n* = 3). (**E**) ATP content was assayed (*P < 0.05 vs. NG+control siRNA, *n* = 4).

To further investigate the role of RCAN1.4 in HG-induced mitochondrial fission and matrix production, RCAN1.4 expression was inhibited using RCAN1.4 siRNA. Under HG conditions, MCs transfected with RCAN1.4 siRNA had an attenuation of mitochondrial fragmentation compared with MCs transfected with control siRNA (Figure 4A,B). At the same time, MCs transfected with RCAN1.4 siRNA had a higher level of Mfn2 and a reduced level of Drp1 in response to HG (Figure 4C). RCAN1.4 siRNA also partly reversed HG-induced decreases of Δ*Ψ*m and ATP content (Figure 4D,E and Supplemenatry Figure S1C). In addition, RCAN1.4 knockdown inhibited HG-induced FN up-regulation (Figure 4C). Taken together, these results indicated that RCAN1.4 inhibition could attenuate mitochondrial fragmentation and matrix production upon HG exposure.

### RCAN1.4-induced matrix production is dependent on its modulation of mitochondrial fission

**Figure 5:**
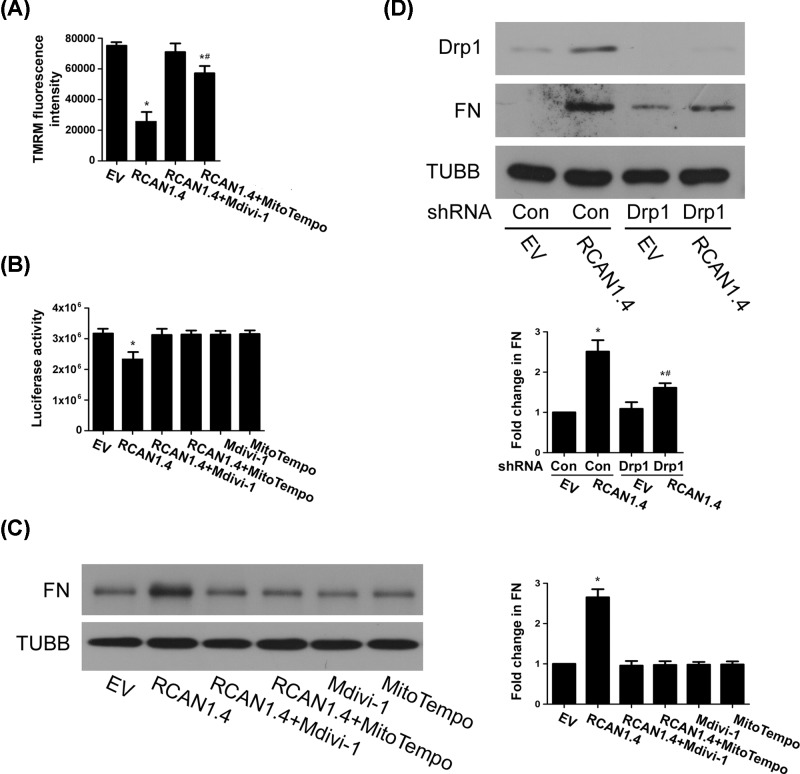
RCAN1.4 overexpression-induced matrix protein up-regulation is partly dependent on mitochondrial fission MCs were pretreated with mitochondrial fission inhibitor Mdivi-1 or mtROS scavenger MitoTEMPO, followed by transfection with RCAN1.4 for 24 h. (**A**) Δ*Ψ*m was evaluated by TMRM staining (*P < 0.05 vs. EV, ^#^*P* < 0.05 vs. RCAN1.4, *n* = 3). (**B**) ATP content was assayed (*P < 0.05 vs. EV, *n* = 4). (**C**) Protein levels of FN were detected by Western blots (*P < 0.05 vs. EV, ^#^*P* < 0.05 vs. RCAN1.4, *n* = 4). (**D**) MCs were transfected with control or Drp1 shRNA for 24 h, followed by RCAN1.4 transfection for 24 h, and then protein level of FN was detected by Western blots (*P < 0.05 vs. control shRNA+EV, ^#^*P* < 0.05 vs. control shRNA+RCAN1.4, *n* = 4).

Our results above demonstrated that RCAN1.4 mediates HG-induced mitochondrial fission, ROS generation and matrix production; however, it is unclear whether mitochondrial fission and ROS are involved in matrix production. To further explore the possible mechanisms, a mitochondrial fission inhibitor Mdivi-1 and a mtROS scavenger MitoTEMPO were used in MCs overexpressing RCAN1.4. Pretreatment with Mdivi-1 or MitoTEMPO prevented RCAN1.4-induced decreases of Δ*Ψ*m (Figure 5A and Supplementary Figure S1D,E) and ATP content (Figure 5B), as well as up-regulation of FN (Figure 5C). Moreover, transfection with Drp1 shRNA also inhibited RCAN1.4-induced FN up-regulation (Figure 5D). These results suggested that RCAN1.4-induced matrix production is dependent on mitochondrial fragmentation in MCs.

**Figure 6:**
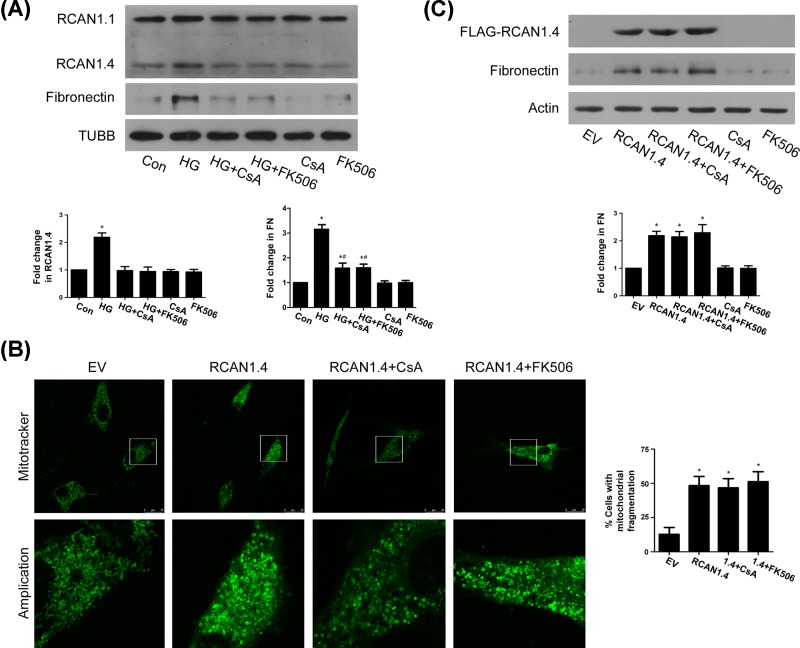
RCAN1.4 overexpression-induced mitochondrial fission and matrix protein upregulation is independent of calcineurin (**A**) MCs were pretreated with calcineurin inhibitor CsA or FK506, followed by incubation with HG for 12 h, and then protein levels of RCAN1.4 and FN were detected by Western blots (*P < 0.05 vs. control, ^#^*P* < 0.05 vs. HG, *n* = 5). (**B** and **C**) MCs were pretreated with CsA or FK506, followed by RCAN1.4 transfection for 24 h. Mitochondrial morphology (**B**) was assessed by Mitotracker-Green staining (*P < 0.05 vs. EV). Protein level of FN (**C**) was detected by Western blots (*P < 0.05 vs. EV, *n* = 3).

Calcineurin has been reported to dephosphorylate Drp1 and mediate mitochondrial fission [28]. As an endogenous regulator of calcineurin [13], RCAN1.4-induced Drp1 up-regulation and translocation to mitochondria may be affected by calcineurin activity. We therefore treated MCs with calcineurin inhibitor CsA and FK506. Both inhibitors blocked HG-induced RCAN1.4 and FN up-regulation (Figure 6A), but had little effect on RCAN1.4 overexpression-induced mitochondrial fission (Figure 6B) and FN up-regulation (Figure 6C). These results indicated that although HG-induced RCAN1.4 up-regulation is dependent on calcineurin activity, RCAN1.4-mediated mitochondrial fission and matrix production might be independent of calcineurin.

## Discussion

In the present study, we demonstrate that HG induces an isoform-specific increase in mRNA and protein expression of RCAN1.4 in rat MCs. RCAN1.4-mediated mitochondrial fission contributes to increased matrix production in response to HG.

One of the main pathological features of DKD is glomerulosclerosis. As one of the innate cells of glomeruli, MCs play an important role in the pathogenesis of DKD. Exposure of MCs to HG can induce proliferation, hypertrophy and increased matrix protein synthesis although the exact mechanisms remain to be clarified. Recent studies have delineated a key role of mitochondrial dysfunction in acute or chronic tubular injury [29–31]; however, studies in MCs are somewhat limited. We therefore investigated the underlying mechanism of HG-induced mitochondrial dysfunction in MCs and further ascertain whether it has an impact on matrix protein synthesis.

Several lines of evidence suggest that RCAN1 is involved in the regulation of mitochondrial function in different cell types [19–21]. However, the role of RCAN1 in renal cells is still unknown. Therefore, we try to investigate whether HG alters the expression levels of RCAN1.1 and RCAN1.4 in glomerular MCs, and whether RCAN1.1 and RCAN1.4 are involved in HG-induced mitochondrial dysfunction. We found that rat MCs have higher basal protein level of RCAN1.1 compared with RCAN1.4. HG induced the up-regulation of RCAN1.4 protein rather than RCAN1.1 protein, which is consistent with previous studies showed that RCAN1.1 is constitutively expressed in most issues, while RCAN1.4 transcription is induced *de novo* by several stimuli [32–35]. In addition, the mRNA level of RCAN1.4 rose in the early stage of HG stimulation and fell 12 h later, while the protein level of RCAN1.4 remained high at 36 h. The discrepancy between RCAN1.4 mRNA and protein levels suggested that there may be translational or post-translational regulation. The ambient level of a protein is the result of rate of synthesis and rate of degradation; therefore, more studies are needed to explore the mechanisms of mRNA translation or protein degradation of RCAN1.

Recent studies showed that overexpression of RCAN 1.1 in β cells can lead to impaired mitochondrial function [19]. RCAN1 overexpression also promotes age-dependent mitochondrial dysregulation and progressive neurodegeneration in Alzheimer’s disease [20]. On the other hand, mitochondrial function is reduced in cardiomyocytes depleted of RCAN1, and increasing RCAN1.1 level helps maintain a more fused mitochondrial network and increase O_2_ consumption [21]. These contrary regulatory effects suggest that more in-depth studies are needed on how RCAN1 works. Our data showed that neither RCAN1.1 nor RCAN1.4 translocated to mitochondria upon HG stimulation in rat MCs; however, RCAN 1.4 up-regulation participated in mitochondrial fission. Overexpression of RCAN1.4 reduced the protein level of MOM profusion protein Mfn2, induced increased the expression of mitochondrial profission protein Drp1 and translocation to mitochondria, eventually leads to mitochondrial fission under NG or HG conditions. Furthermore, inhibition of RCAN1.4 expression by siRNA prevented HG-induced Drp1 up-regulation and mitochondrial fragmentation, indicating that RCAN1.4 plays a key role in mitochondrial fission in the states of hyperglycemia. Further research is needed to elucidate the exact mechanism of RCAN1.4-induced up-regulation and translocation of Drp1 to mitochondria.

Mitochondrial fission is commonly related to increased mtROS generation [26,27]. Yu et al. showed rapid mitochondrial fragmentation with concomitant increased mtROS in rat liver cells as a result of HG exposure. Moreover, inhibition of mitochondrial fission prevented HG-induced ROS generation in several cell types [27,36]. Mitochondrial fragmentation and increased mtROS are often associated with a decrease in mitochondrial function, resulting in reduced ATP production [37]. Consist with this, our data showed that MCs overexpressing RCAN1.4 had decreased mitochondrial membrane potential and ATP content, but mtROS production was increased. RCAN1.4 knockdown by siRNA attenuated HG-induced mitochondrial fragmentation, therefore restored mitochondrial function, mainly reflected in the increase of mitochondrial membrane potential and ATP production.

Increasing evidence indicates that disturbances in mitochondrial homeostasis are important in the development and progression of DKD. Mitochondrial dysfunction activates abnormal signals such as oxidative stress and apoptosis in different cell types under HG ambience [4,34]; however, the relationship between mitochondrial fission and increased matrix production is still poorly understood. Our results showed that RCAN 1.4 mediates HG-induced matrix production, which can be prevented by mitochondrial fission inhibitor Mdivi-1 and Drp1 shRNA, indicating that RCAN1.4-induced matrix production is dependent on its modulation of mitochondrial fragmentation. Although the underlying mechanism is unclear, one possible explanation is that mitochondrial fission leads to increased intracellular ROS, which may enhance ECM protein production. Oxidative stress is generally thought to play an important role in diabetic renal injury. ROS can activate several signal transduction cascade (such as PKC, MAPK and JAK-STAT pathway) and downstream transcription factors to up-regulate ECM genes and proteins in glomerular MCs [38–40]. Our data showed that mtROS scavenger MitoTEMPO also inhibited RCAN1.4-induced FN up-regulation, which strongly suggested a direct role of ROS in overproduction of ECM proteins.

It is becoming increasingly evident that calcineurin is activated in diabetes and contributes to matrix overproduction in renal MCs [41,42]. As an endogenous inhibitor of calcineurin, RCAN1.4 overexpression was supposed to prevent HG-induced FN up-regulation, as shown by calcineurin inhibitors CsA and FK506. Surprisingly, although HG-induced RCAN1.4 expression is dependent on calcineurin activity, our results showed that RCAN1.4 overexpression increased matrix production in rat MCs. The possible mechanism is that continuous overexpression of RCAN1.4 has a detrimental effect on MCs, which is independent of calcineurin. Recent studies also support the notion that although the short-term induction of RCAN1 expression generally has a protective effect in multiple cell types by inhibiting the calcineurin/NFAT transcriptional pathway [16,43], chronic overexpression of RCAN1 may drive pathophysiological changes in neurons and endocrine cells linked to Down syndrome, Alzheimer’s disease and Type 2 diabetes [17,44,45].

In conclusion, the present study shows that RCAN1.4 up-regulation under HG conditions induces mitochondrial fission and dysfunction, which mediates extracellular matrix production in rat MCs. Therefore, inhibition of RCAN1.4 might be a therapeutic potential for prevention of glomerulosclerosis in DKD.

## Supplementary Material

Supplementary Figure S1

## Abbreviations

Δ*Ψ*m: mitochondrial membrane potential
CCCP: carbonyl cyanide m-chlorophenylhydrazone
Col I: collagen I
CsA: Cyclosporine A
DKD: Diabetic kidney disease
Drp1: Dynamin related protein 1
EV: empty vector
Fis1: mitochondrial fission 1 protein
FN: fibronectin
HG: high glucose
MC: mesangial cell
Mfn2: mitofusin 2
mtROS: mitochondrial reactive oxygen species
NG: normal glucose
Opa1: optic atrophy 1
RCAN1: regulators of calcineurin 1
TMRM: tetramethylrhodamine methyl ester
