# Supplementary material for: RCAN1.4 mediates high glucose-induced matrix production by stimulating mitochondrial fission in mesangial cells
Source: Biosci Rep. 2020 Jan 17;40(1):BSR20192759. doi: 10.1042/BSR20192759 (PMC6970086; doi:10.1042/BSR20192759)
Supplement: Supplementary Figure S1 [file BSR-2019-2759_supp.pdf]

Suppl.fig. 1

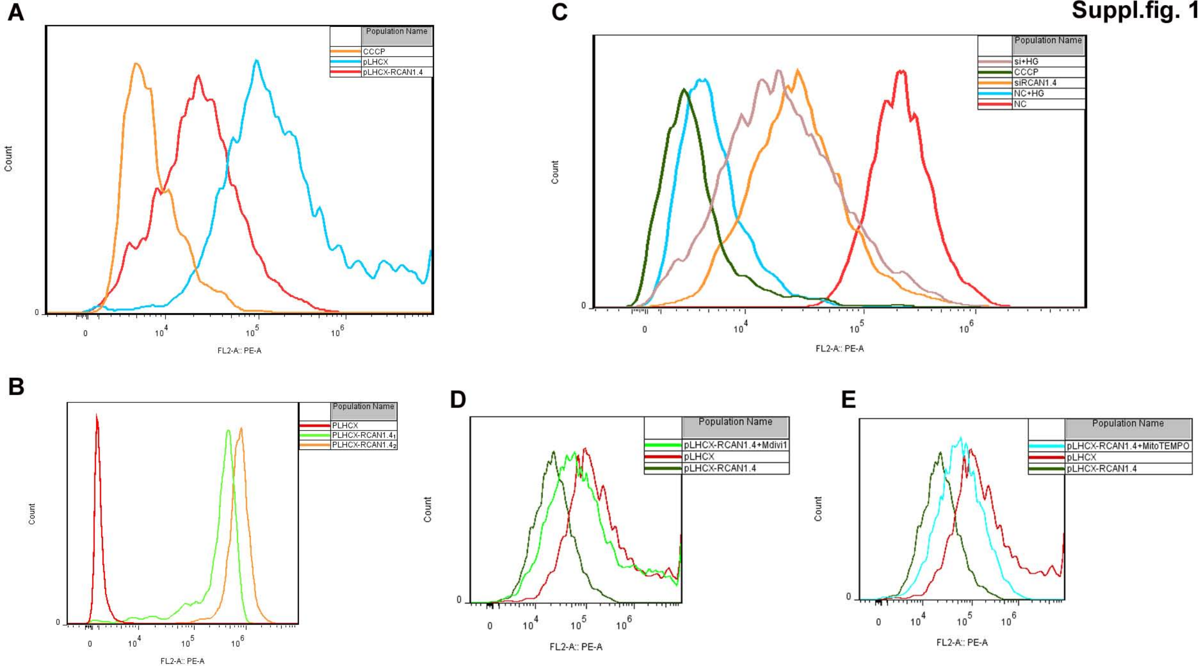
A
Population Name
CCCP
pLHCX
pLHCX-RCAN1.4
Count
FL2-A:: PE-A
B
Population Name
PLHCX
PLHCX-RCAN1.4₁
PLHCX-RCAN1.4₂
Count
FL2-A:: PE-A
C
Population Name
si+HG
CCCP
siRCAN1.4
NC+HG
NC
Count
FL2-A:: PE-A
D
Population Name
pLHCX-RCAN1.4+Mdivi1
pLHCX
pLHCX-RCAN1.4
Count
FL2-A:: PE-A
E
Population Name
pLHCX-RCAN1.4+MitoTEMPO
pLHCX
pLHCX-RCAN1.4
Count
FL2-A:: PE-A

Supplementary Figure Legends

Fig.1 Flow cytometry analysis of mitochondrial membrane potential (Δ*Ψ*m) and mitochondrial reactive oxygen species (mtROS). Representative graphs of fluorescence intensity (TMRM: excitation/emission 543/580 nm, MitoSOX Red: excitation/emission 510/590 nm) were shown. A: MCs was transfected by pLHCX-RCAN1.4 or empty vector, ΔΨm was evaluated by TMRM staining with CCCP as positive control. B: MCs was transfected by pLHCX-RCAN1.4 for 24h (1) or 48 h (2), and then mtROS was evaluated by MitoSOX Red staining. C: After negative control (NC) or RCAN1.4 siRNA transfection for 36 h, MCs were incubated with HG for 24 h. Δ*Ψ*m was evaluated by TMRM staining with CCCP as positive control. D, E: MCs were pretreated with Mdivi-1 or MitoTEMPO, followed by transfection with pLHCX-RCAN1.4 or empty vector for 24 h. Δ*Ψ*m was evaluated by TMRM staining.
